# Supplementary material for: Reducing mitochondrial reads in ATAC-seq using CRISPR/Cas9
Source: Sci Rep. 2017 May 26;7:2451. doi: 10.1038/s41598-017-02547-w (PMC5446398; doi:10.1038/s41598-017-02547-w)
Supplement: Supplementary file 1 — Supplemental figures and tables [file 41598_2017_2547_MOESM1_ESM.pdf]

Supplemental Material - Reducing mitochondrial reads in ATAC-seq using CRISPR/Cas9

Lindsey Montefiori, Liana Hernandez, Zijie Zhang, Yoav Gilad, Carole Ober, Gregory Crawford, Marcelo Nobrega, Noboru Jo Sakabe

Fold-differences in Supplemental Figs. 1 and 2 were calculated between the highest and lowest medians. Paired one-tailed Wilcoxon tests were performed between DT x DT samples and ND x ND samples. Unpaired tests were performed between DT and ND samples.

Supplemental Figure S1 – Number of peaks identified with HOMER and MACS2 using different parameters and 9.8 M, 17 M and 21.9 M reads. Compare to Figure 3c of the main text. The plot shown in the main text is shown in a frame.

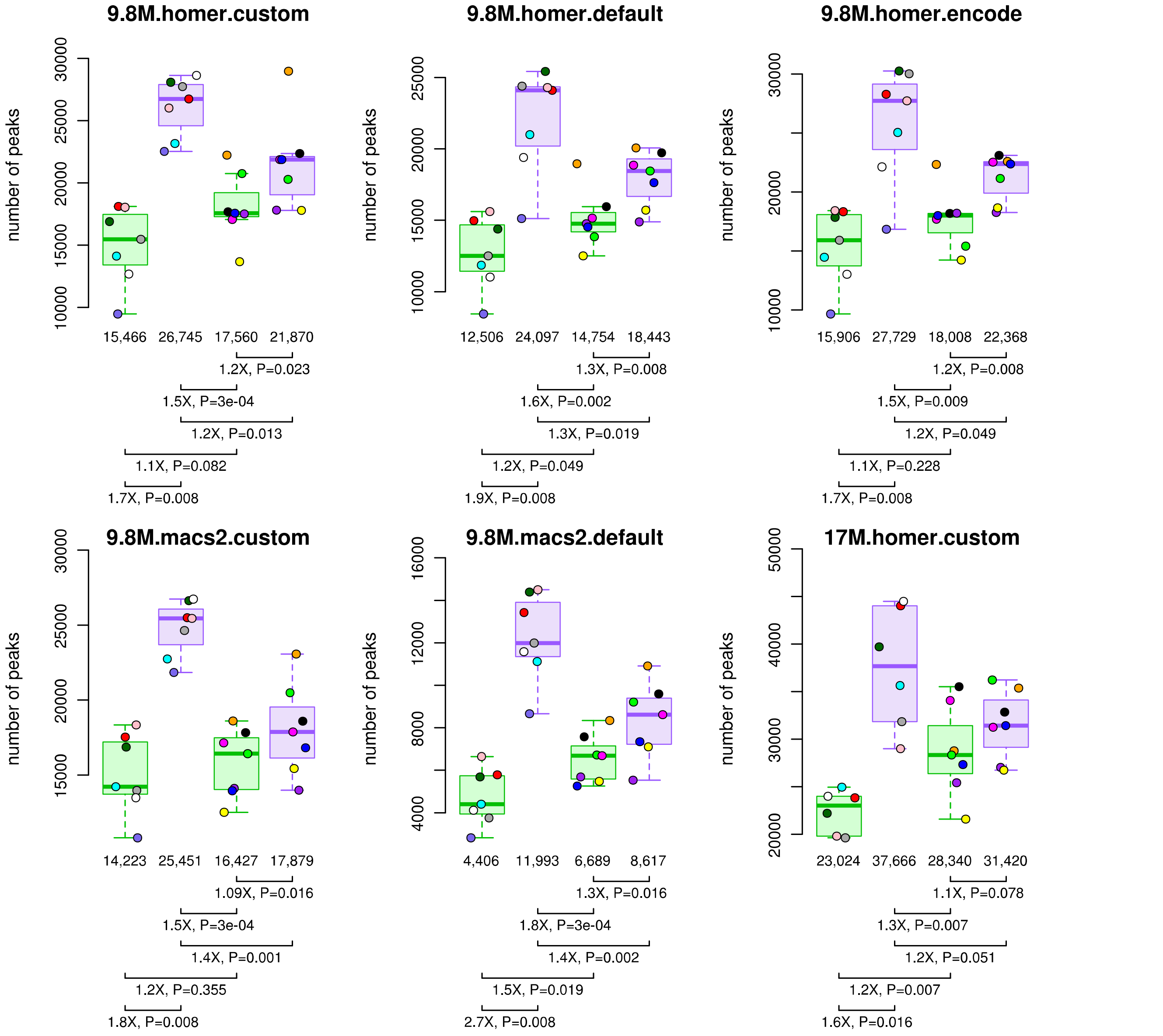

Continuation of Fig S1.

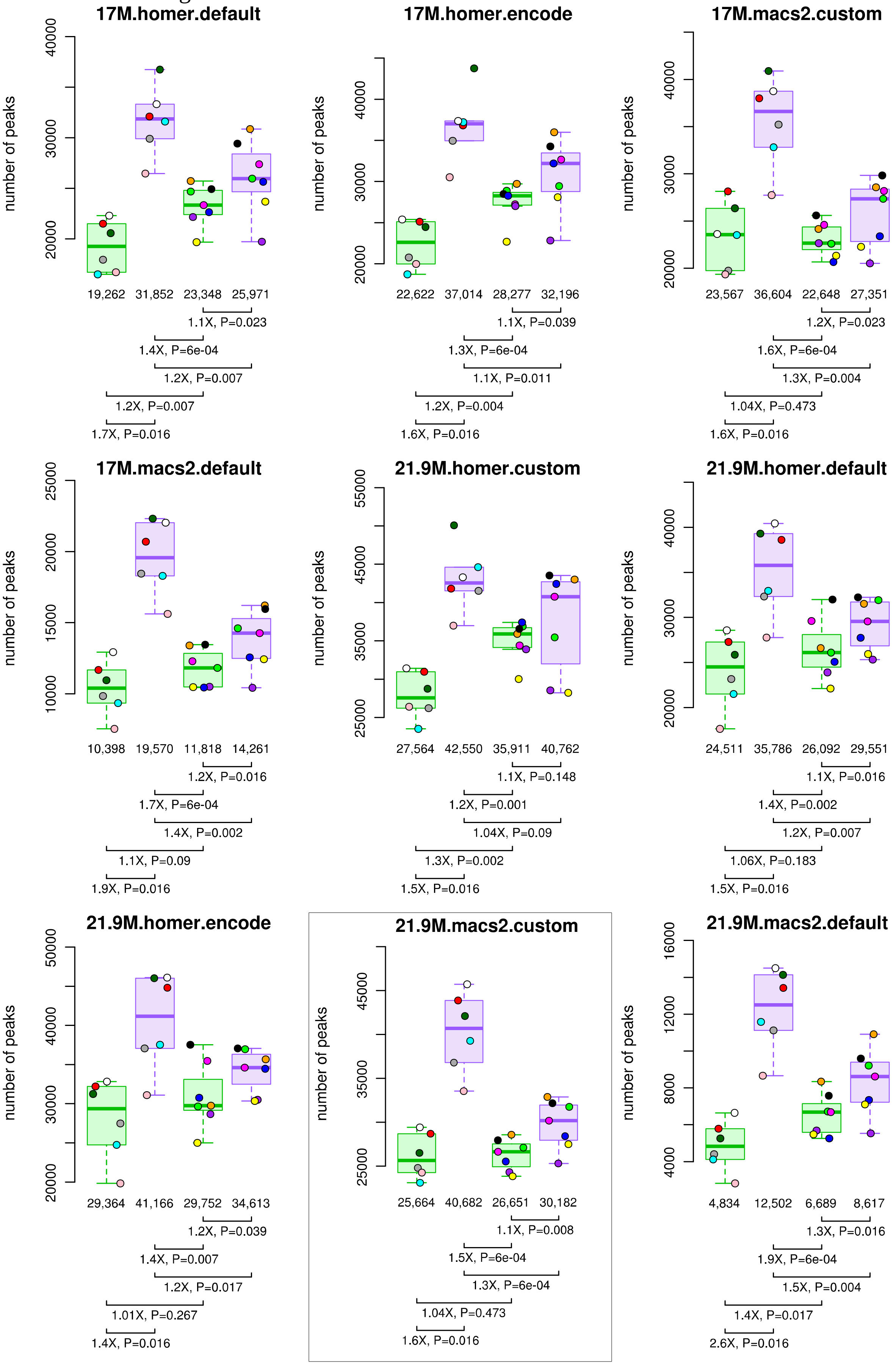

Supplemental Figure S2 – Fraction of peaks overlapping Epigenome Roadmap lymphoblastoid cell enhancers. Compare to Figure 4b of the main text. The plot shown in the main text is shown in a frame.

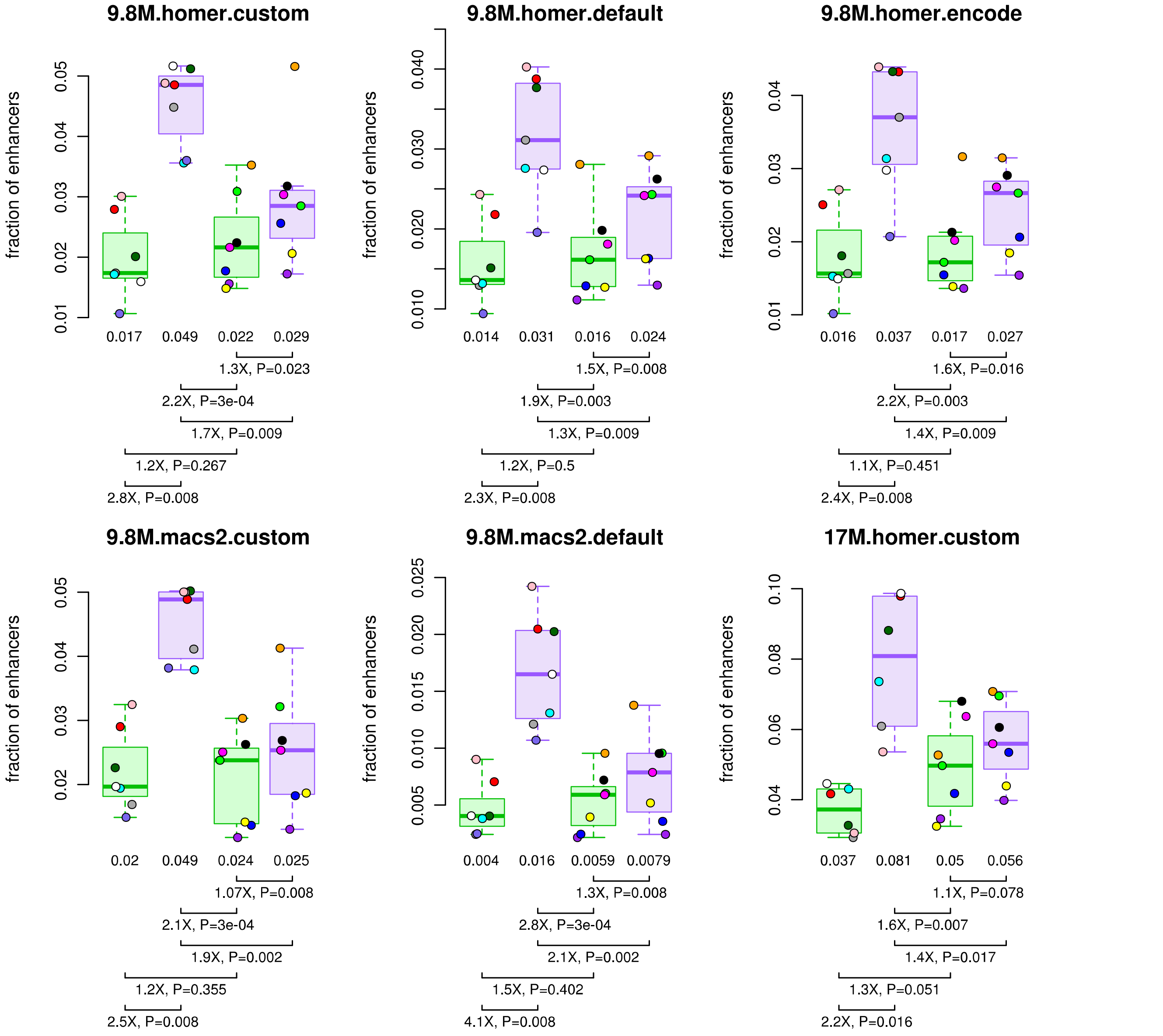

Continuation of Fig. S2

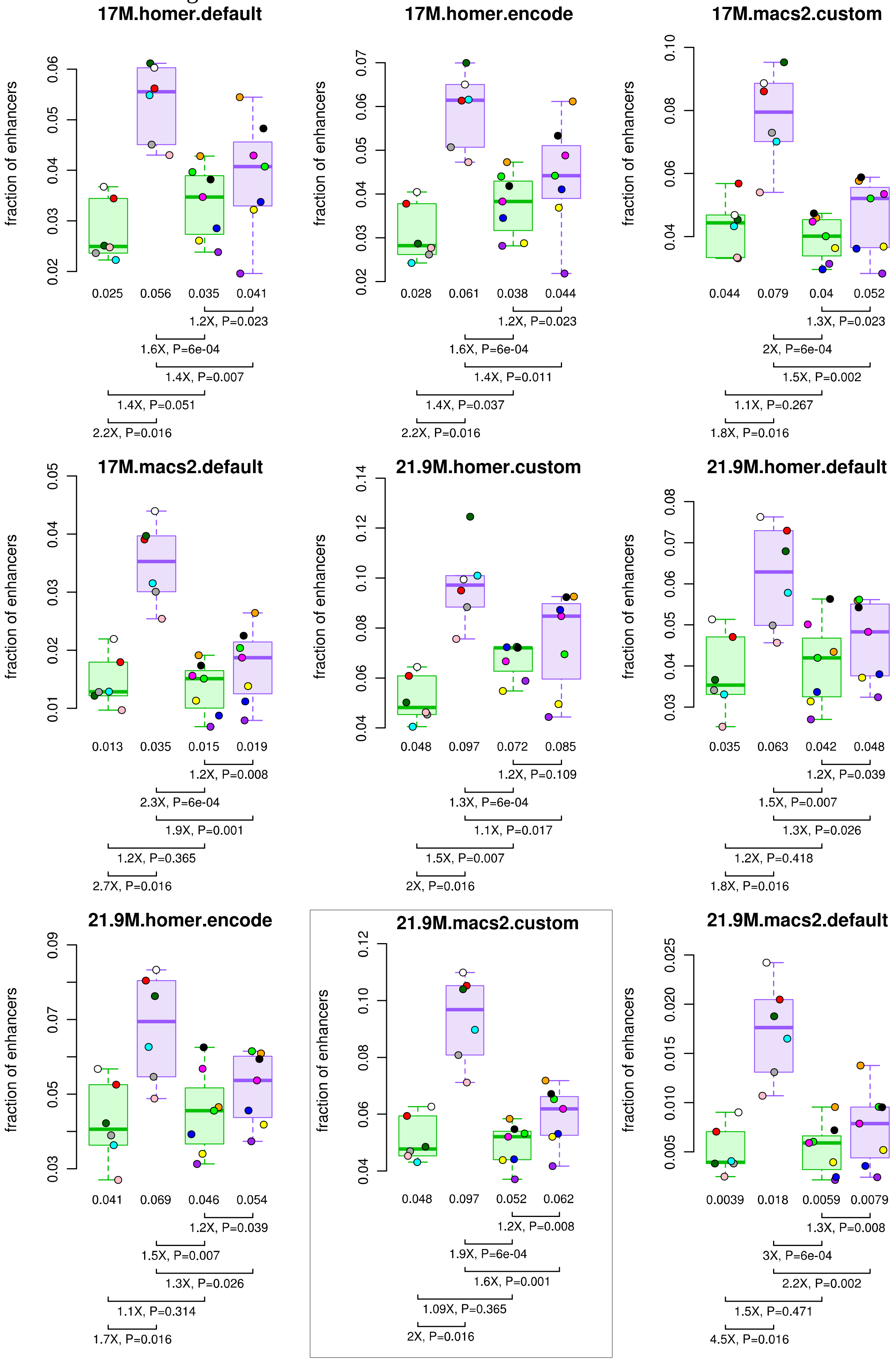

Supplemental Table S1 – Summary of fold-differences between the number of peaks called in anti-mt CRISPR treated (TR) and untreated samples (UN), and samples prepared with (DT) and without detergent (ND) in the cell lysis buffer. Data from Supplementary Fig. 1.

	UN-DT x TR-DT	UN-DT x UN-ND	TR-DT X TR-ND	TR-DT X UN-ND	UN-ND x TR-ND
9.8M-homer-custom	1.7	1.1	1.2	1.5	1.2
9.8M-homer-default	1.9	1.2	1.3	1.6	1.3
9.8M-homer-encode	1.7	1.1	1.2	1.5	1.2
9.8M-macs2-custom	1.8	1.2	1.4	1.5	1.09
9.8M-macs2-default	2.7	1.5	1.4	1.8	1.3
17M-homer-custom	1.6	1.2	1.2	1.3	1.1
17M-homer-default	1.7	1.2	1.2	1.4	1.1
17M-homer-encode	1.6	1.2	1.1	1.3	1.1
17M-macs2-custom	1.6	0.96	1.3	1.6	1.2
17M-macs2-default	1.9	1.1	1.4	1.7	1.2
21.9M-homer-custom	1.5	1.3	1.04	1.2	1.1
21.9M-homer-default	1.5	1.06	1.2	1.4	1.1
21.9M-homer-encode	1.4	1.01	1.2	1.4	1.2
21.9M-macs2-custom	1.6	1.04	1.3	1.5	1.1
21.9M-macs2-default	2.6	1.4	1.5	1.9	1.3
mean	1.8	1.2	1.3	1.5	1.2
stdev	0.4	0.1	0.1	0.2	0.1

Supplemental Table S2 – Fold-differences between the fraction of enhancers identified in anti-mt CRISPR treated (TR) and untreated samples (UN), and samples prepared with (DT) and without detergent (ND) in the cell lysis buffer. Data from Supplementary Fig. 2.

	TR-DT X UN-ND	UN-ND x TR-ND
9.8M-homer-custom	2.2	1.3
9.8M-homer-default	1.9	1.5
9.8M-homer-encode	2.2	1.6
9.8M-macs2-custom	2.1	1.07
9.8M-macs2-default	2.8	1.3
17M-homer-custom	1.6	1.1
17M-homer-default	1.6	1.2
17M-homer-encode	1.6	1.2
17M-macs2-custom	2	1.3
17M-macs2-default	2.3	1.2
21.9M-homer-custom	1.3	1.2
21.9M-homer-default	1.5	1.2
21.9M-homer-encode	1.5	1.2
21.9M-macs2-custom	1.9	1.2
21.9M-macs2-default	3	1.3
mean	2.0	1.3
stdev	0.5	0.1

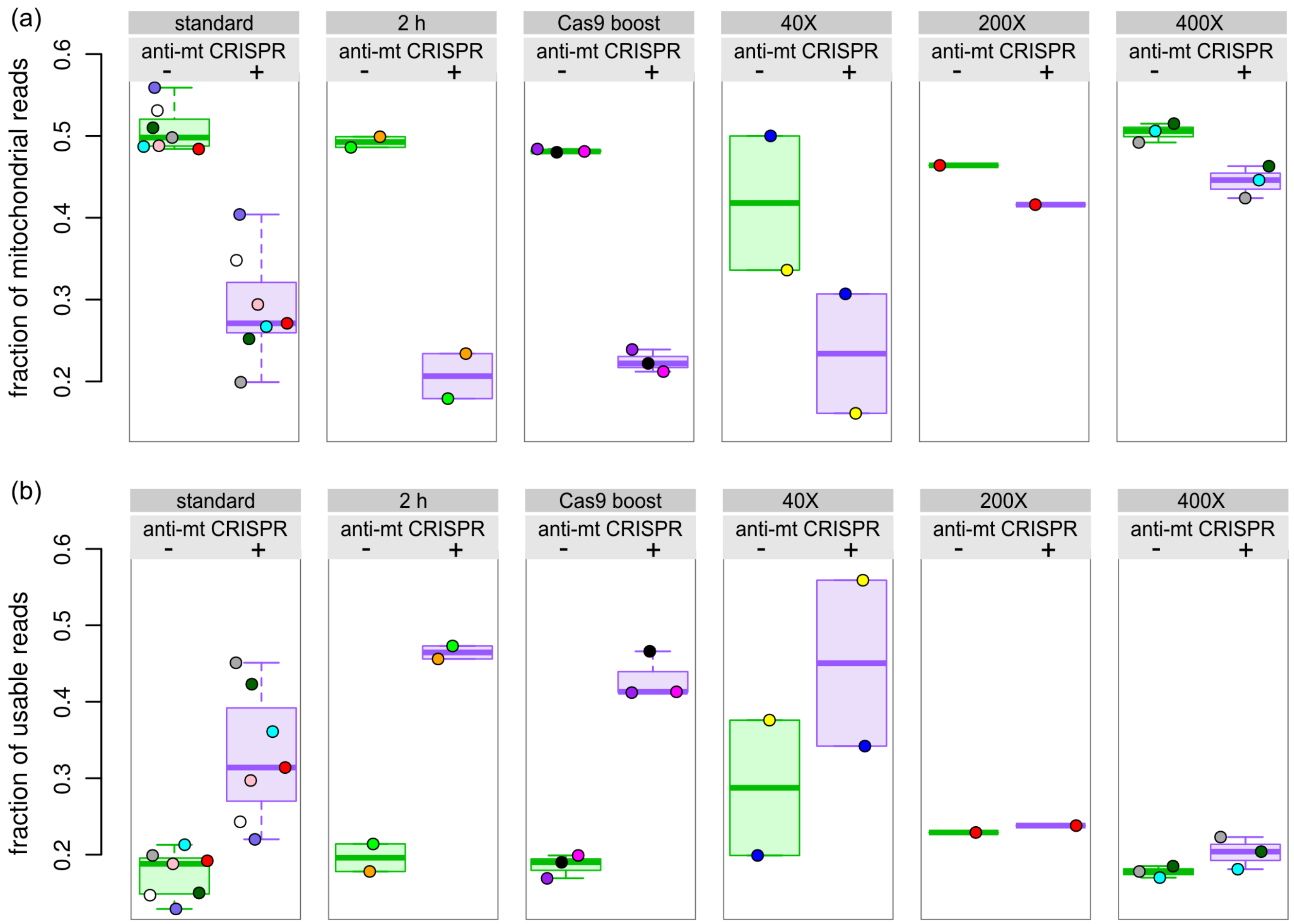

Supplemental figure S3 – Effect of modifications of the anti-mt CRISPR/Cas9 treatment on the fraction of mitochondrial reads and usable reads. The number of peaks is shown in Figure 5.

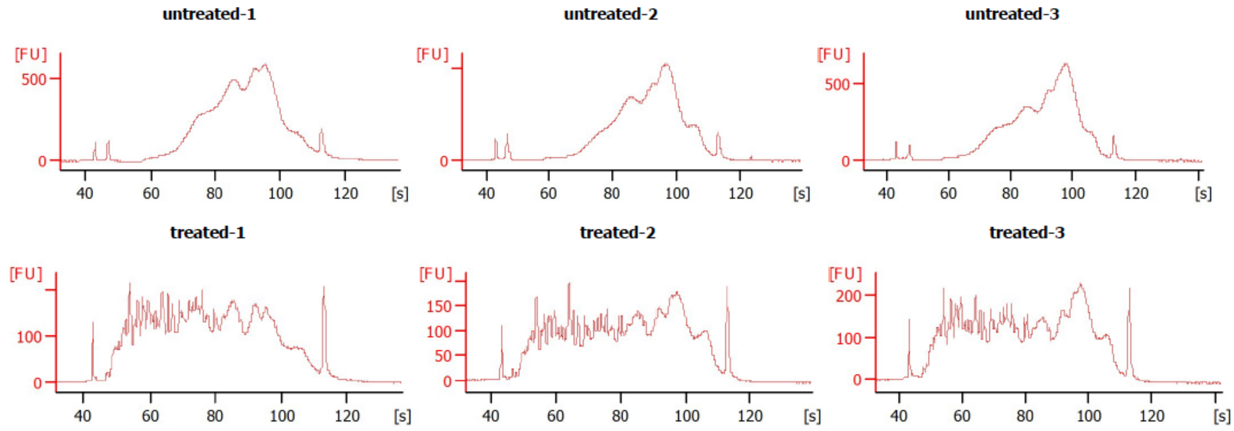

Supplemental figure S4 – High sensitivity Bionalyzer traces showing 3 replicates of ATAC-seq libraries before and after CRISPR/Cas9 treatment. The X-axis shows the number of seconds that takes the fragments to move through the channel – the longer the fragment, the longer the time. Note the increase in small fragments in the treated samples which theoretically corresponds to cleaved mtDNA sequences.

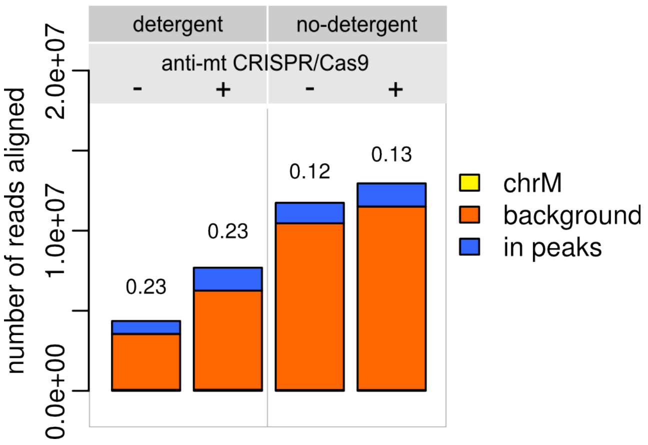

Supplemental Figure S5 – Background is higher in ND samples. This figure is similar to Figure 3d of the main text but using unique reads. The fraction of chrM reads is too small to be displayed. ND samples have higher background than DT samples.

Estimation of multi-mapping reads

We aligned all reads with bowtie2 (2.2.3) with default parameters with the exception of setting -k 2. We then extracted all reads with MAPQ = 1 and counted the number of reads aligned to chrM and any other nuclear chromosome. We divided the number obtained by the total number of reads sequenced as an estimate of the number of reads discarded due to alignment to other locations in addition to aligning to the mitochondrial genome.
